# ESPO and Behavioral and Social Sciences Section Symposium: Innovative Approaches to Advancing Environmental Justice in Aging Research

**DOI:** 10.1093/geroni/igaf122.541

**Published:** 2025-12-31

**Authors:** Monica Walters, Eun Young Choi

## Abstract

Environmental justice is increasingly recognized as an important dimension of aging research, particularly in understanding later-life health disparities. Structural inequities in environmental exposures disproportionately affect racially and ethnically minoritized older adults, exacerbating disparities in cognitive, mental, and functional health. However, critical gaps remain in identifying what specific environmental determinants contribute to these inequities and the mechanisms through which they operate. This symposium brings together cutting-edge conceptual and methodological approaches to advance environmental justice in aging research. The first three presentations address neighborhood-driven disparities in cognitive aging. Dr. Esposito uses a novel sequential mixed-methods approach to critically consider how conceptualizations of “place” (e.g., neighborhood risk/protective factors) shape cognitive outcomes. Dr. Muñoz investigates the temporal effects of neighborhood exposures at macro- and micro-time scales on cognition, drawing on multiple datasets including racially and ethnically diverse populations. Dr. Abdel Magid assesses the relative contributions of different spatial social polarization domains to dementia risk, utilizing Medicare claims data. The final two presentations focus on environmental hazards and racism as structural determinants of health inequities. Ms. Holland assesses relationships between extreme heat exposure and depression risk among older Mexican Americans, leveraging data from the Hispanic Established Population for the Epidemiological Study of the Elderly. Dr. Adkins-Jackson examines how early-life and adulthood exposure to environmental racism shapes late-life functional abilities among Black residents, using the Behavioral Risk Factor Surveillance System. Together, these presentations will advance understanding of how environmental exposures contribute to health disparities, pointing to policy changes that can promote health equity.

